# Accumulation of connective tissue growth factor^+^ cells during the early phase of rat traumatic brain injury

**DOI:** 10.1186/1746-1596-9-141

**Published:** 2014-07-10

**Authors:** Yuqi Liu, Zongwei Liu, Xiaoming Li, Bangwei Luo, Jian Xiong, Woting Gan, Man Jiang, Zhiyuan Zhang, Hermann J Schluesener, Zhiren Zhang

**Affiliations:** 1 Institute of Immunology, Third Military Medical University, 30 Gaotanyan Main Street, Chongqing 400038, People’s Republic of China; 2 Institute of Brain Research, University of Tuebingen, Tuebingen, Germany

**Keywords:** Connective tissue growth factor, Astrocytes, Weight-drop model, Traumatic brain injury

## Abstract

**Abstract:**

**Background**

Glial scar formation is a common histopathological feature of traumatic brain injury (TBI). Astrogliosis and expression of transforming growth factor beta (TGF-β) are key components of scar formation and blood-brain barrier modulation. Connective tissue growth factor (CTGF) is considered a cytokine mediating the effects of TGF-β.

**Methods**

Here, we studied the CTGF expression in an open-skull weight-drop-induced TBI, with a focus on the early phase, most amenable to therapy.

**Results**

In normal rat brains of our study, CTGF^+^ cells were rarely observed. Significant parenchymal accumulation of CTGF^+^ non-neuron cells was observed 72 h post-TBI and increased continuously during the investigating time. We also observed that the accumulated CTGF^+^ non-neuron cells were mainly distributed in the perilesional areas and showed activated astrocyte phenotypes with typical stellate morphologic characteristics.

**Conclusion**

Our observations demonstrated the time-dependent and lesion-associated accumulation of cellular CTGF expression in TBI, suggesting a pathological role of CTGF in TBI.

**Virtual Slides:**

The virtual slide(s) for this article can be found here: http://www.diagnosticpathology.diagnomx.eu/vs/3963462091241165

## Background

Glial scar formation is a common histopathological feature of traumatic brain injury (TBI). It persists for long periods and acts as barrier not only to axon regeneration but also to inflammatory cells in a manner that protects healthy tissue from nearby areas of intense inflammation [1]. Reactive astrocytosis is the key contributes to glial scar formation [2]. Induced by stimuli from lesions, astrocytes enter into the injured zone, are activated, and play a major role in the response to TBI. In central nervous system (CNS) injuries, activated astrocytes secrete many cytokines, among which transforming growth factor (TGF)-β is regarded as one of the most potent cytokines, it can be selectively upregulated in astrocytes and contribute to scar formation after injury through inducing secretion of another “downstream mediator”, the cytokine connective tissue growth factor (CTGF) [3,4].

CTGF is a secreted peptide encoded by an immediate early growth responsive gene that has been shown to be a downstream mediator of TGF-β1 action, induces mitogenesis, chemotaxis, and cell matrix induction of fibroblasts [5]. Due to these properties, CTGF plays important roles in the regulation of scar formation, wound healing, cell migration, proliferation and extracellular matrix [4,6]. A series of studies has also demonstrated that CTGF over-expression correlates with many fibrotic and inflammation-associated diseases, such as fibrotic skin disease, atherosclerosis and inflammatory bowel disease [7-9].

Upregulation of CTGF has been observed in a variety of nervous system-related disorders. In Alzheimer’s disease, CTGF was observed to overexpress in perivascular astrocytes and in astrocytes associated with plaques, indicating the role for CTGF in the process of chronic neurodegeneration [10]. In amyotrophic lateral sclerosis, CTGF was dramatically increased in reactive astrocytes of the ventral horn, supporting a role for CTGF in the molecular mechanisms underlying astrogliosis [11]. Although the expression of CTGF has been reported in human TBI, mice spinal cord injury, and rat kainic acid-induced brain injury, altered CTGF expression following TBI models are not completely clear. Therefore, in the current study, we have investigated the spatiotemporal expression of CTGF following an open-skull weight-drop-induced TBI in rat brains.

## Methods

### Animal experiments and tissue library

Brain libraries of normal and TBI rats have been described previously [12]. In brief, male Lewis rats (8–9 weeks of age, 350–400 g; Elevage Janvier, Le Genest-St-Isle, France) were housed under equal daily periods of light and dark and with free access to food and water. All procedures were performed in accordance with the published International Health Guidelines under a protocol approved by the Administration District Official Committee. The number of rats used and their suffering were minimized. TBI was induced in anesthetized rats using an open-skull weight-drop contusion model. Rats were grouped randomly, anesthetized with Ketamine (120 mg/kg)/Rompun (8 mg/kg), and subjected to craniotomy, in which a circular region of the skull (3.0 mm diameter, centered 2.3 mm caudal and 2.3 mm lateral to bregma) was removed over the right somatosensory cortex. A weight-drop device was placed over the dura and adjusted to stop an impact transducer (foot plate) at a depth of 2.5 mm below the dura. Then, a 20 g weight was dropped from 15 cm above the dura, through a guiding tube onto the foot plate. Body temperature was maintained using an overhead heating lamp during surgery. After injury, the scalp was closed tightly. Rats with TBI survived without further treatment and were euthanized at different time points (6, 12, 18, 24, 48, 72, and 96 h; 3–5 rats/time point).

For euthanasia, rats (26 TBI rats, five normal control rats) were deeply anesthetized with Ketamine (120 mg/kg)/Rompun (8 mg/kg) and perfused intracardially with 4°C 4% paraformaldehyde in PBS. Brains were quickly removed and postfixed in 4% paraformaldehyde overnight at 4°C. Fixed rat whole brains were placed in rodent brain matrices (coronal) and were sliced to obtain the cortical coronal blocks containing the contusion regions. These cortical coronal blocks were embedded in paraffin, serially sectioned (3 μm) through the center of the traumatized area, and mounted on silan-covered slides. The contused areas were numbered and during the subsequent immunostaining, the same antibody was applied to sections with the same number.

### Immunohistochemistry

After dewaxing, brain sections were boiled (in an 850 W microwave oven) for 15 min in citrate buffer (2.1 g citric acid monohydrate/l, pH = 6) (Carl Roth, Karlsruhe, Germany). Endogenous peroxidase was inhibited by 1% H_2_O_2_ in pure methanol (Merck, Darmstadt, Germany) for 15 min. Sections were incubated with 10% normal pig serum (Biochrom, Berlin, Germany) to block nonspecific binding of immunoglobulins and then with the polyclonal antibody CTGF (1:100, BMA; USbio, Swampscott, Massachusetts, USA) overnight at 4°C. Antibody binding to tissue sections was visualized with a biotinylated rabbit anti-mouse IgG F(ab)_2_ antibody fragment (1 : 400; Dako, Hamburg, Germany). Subsequently, sections were incubated with a horseradish peroxidase-conjugated streptavidin complex for 30 min (1: 100; Dako), followed by development with diaminobenzidine substrate (Fluka, Neu-Ulm, Germany). Finally, sections were counterstained with Mayer’s hemalaun. As negative controls, the primary antibodies were excluded.

### Data acquisition and statistical analysis

After immunostaining, the brain sections of each time point were examined by light microscopy. The numbers of CTGF^+^ non-neuron cells were counted in eight nonoverlapping high-power fields (×400 magnification) for each section. The high-power fields were selected to have a maximum of positive cells. In each field studied, only positive cells with the nucleus at the focal plane were counted. Results were presented as arithmetic means of CTGF^+^ non-neuron cells per high-power fields and SEM.

Statistical analysis was carried out using one-way analysis of variance, followed by Dunnett’s multiple comparison tests or a nonparametric t-test (GraphPad Prism 4.0 Software, San Diego, USA). For all statistical analyses, significance levels were set at P value less than 0.05.

## Results

A reproducible local lesion is formed on open-skull weight-drop injury in the ipsilateral cortex. The development of the lesion following TBI was analyzed using H&E staining has been described previously [13]. In brief, selective neuronal loss and necrotic loci condensed at the edge of impact site were observed 12 h postinjury (p.i.). Significant leukocyte infiltration and hemorrhage were already observed on day 1. At day 4 post-TBI, a lesioned cavity together with its surrounding perilesional tissue loss was formed under the impact areas. At the lesional areas, most tissue was lost, no neurons remained, and leukocyte infiltration together with hemorrhage appeared. In the perilesional areas, selective neuron loss with leukocyte infiltration was observed. In the subcortical white matter, no morphological changes were observed.

**Figure 1:**
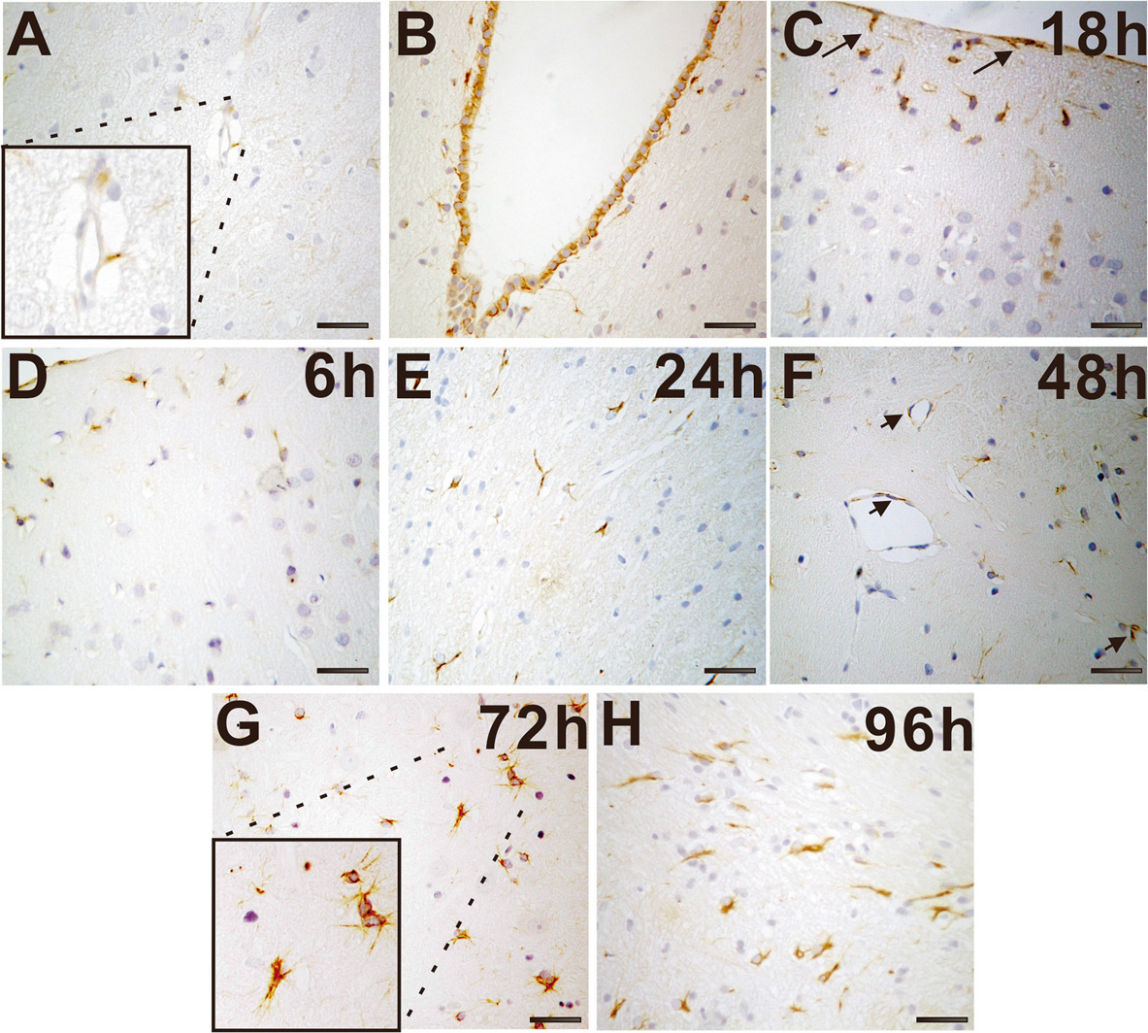
**Accumulation of connective tissue growth factor**^**+ **^**(CTGF**^**+**^**) non-neuron cells in traumatic brain injury. A**: In normal brains, CTGF immunoreactivity was occasionally observed in the brain tissue. The boxed area indicates CTGF immunoreactivity in small vessels. **B**: Almost all ependymal cells showed CTGF^+^. **C-H**: The accumulation of CTGF^+^ cells in the lesioned regions at 18 h **(C)**, 6 h **(D)**, 24 h **(E)**, 48 h **(F)**, 72 h **(G)** and 96 h **(H)**. Arrows indicate the accumulation of CTGF^+^ non-neuron cells with astrocytes phenotype in the cortex and meningeal **(C)**. CTGF immunoreactivity was also detected in endothelial cells **(F)**. A significant increase in CTGF^+^ non-neuron cells was observed confined to the lesion at 72 h postinjury **(G)**. The boxed area indicates the localization of CTGF^+^ non-neuron cells that were mainly identified as astrocytes with typical stellate morphologic characteristics or belt-shaped, grouped pattern. The accumulation of CTGF^+^ non-neuron cells reached the maximum at 96-h postinjury **(H)**. Scale bars are 50 μm.

**Figure 2:**
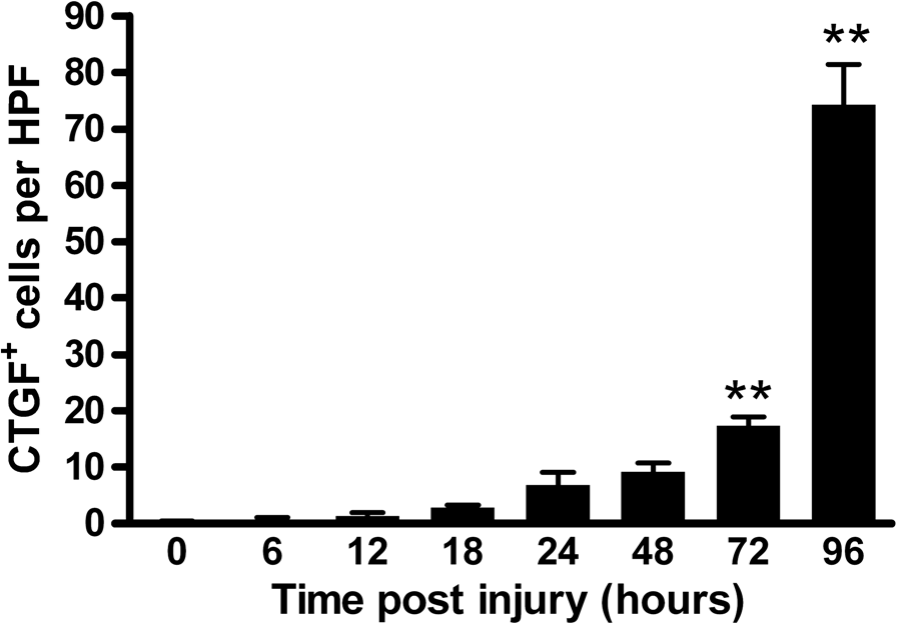
**The time course of parenchymal connective tissue growth factor**^**+ **^**(CTGF**^**+**^**) non-neuron cell accumulation in traumatic brain injury.** The numbers of CTGF^+^ cells of every rat brain were counted in eight high-power fields. We only counted positive cells with the nucleus at the focal plane. Results are presented as arithmetic means of positive cells per high-power field and SEM. Statistical analysis was carried out using one-way analysis of variance, followed by Dunnett’s multiple comparison test (GraphPad Prism 4.0 Software). **P < 0.01 compared with the normal control.

The expression of CTGF post-TBI was studied using immunohistochemistry. In normal adult rat brain, CTGF immunoreactivity was rarely observed in the cortex and meningeal (0.20 ± 0.194 per high-power field, Figures 1A and 2), only a few CTGF immunoreactivity was demonstrated in small vessels (Figure 1A, box indicated). Furthermore, almost all ependymal cells showed CTGF^+^ (Figure 1B). After TBI, no visible change in CTGF immunoreactivity was detected in the contralateral hemisphere (data not shown). In the injured (ipsilateral) sides, we found that CTGF expression in neurons did not change significantly, but the accumulation of non-neuron CTGF^+^ cells was observed in the lesion and perilesional areas (Figure 1C, arrows indicated). We then quantified the non-neuron CTGF^+^ cells in lesional areas. CTGF^+^ neurons were not counted. After TBI, a slight increase in CTGF^+^ non-neuron cells was observed confined to the lesion as early as 6 h p.i. (0.50 ± 0.497 per high-power field, P > 0.05, Figures 1D and 2). The accumulation of CTGF^+^ non-neuron cells increased over time (Figures 1E, F and 2). The accumulation of CTGF^+^ non-neuron cells became significant at 72-h post-TBI (17.40 ± 1.503 per high-power field, P < 0.01, Figures 1G and 2) and reached the maximum at 96-h post-TBI (74.20 ± 7.248, P < 0.01, Figures 1H and 2) during our observed time period.

The accumulation of CTGF^+^ non-neuron cells was mainly in perilesional areas but not in remote areas of the ipsilateral and contralateral hemispheres. At the lesioned site, most of the CTGF^+^ non-neuron cells showed activated astrocytes phenotypes with typical stellate morphologic characteristics or belt-shaped, grouped pattern (Figure 1G, box indicated). CTGF immunoreactivity was also detected in endothelial cells (Figure 1F, arrows indicated), but no significant differences were observed between injured and control brains.

## Discussion

Here we analyzed the accumulation of CTGF^+^ non-neuron cells in rat brain of weight-drop contusion following TBI. Significant CTGF^+^ non-neuron cell accumulation was detected 72-h p.i. and increased markedly. The CTGF^+^ non-neuron cells mainly detected in perilesioned areas where CTGF accumulation various in different time course. Furthermore, most CTGF^+^ non-neuron cells showed activated astrocyte phenotypes with typical stellate morphologic characteristics.

Nowadays, increasingly studies are focusing on the recovery stage after neural tissue injury [14], through which more pro-recovering molecules are discovered [15]. CTGF is investigated as a secreted cell matrix-inducing peptide involved in both tissue regeneration mechanisms [3]. In normal rat brains, CTGF expression can be found in glia-like cells, neurons, endothelial cells and ependymal cells [6]. In the CNS, increased expression of CTGF was induced under certain pathological conditions, such as spinal cord injury and cerebral infarction [6,16]. It was demonstrated that in kainic acid-induced brain injury, significantly increasing of CTGF mRNA expression was limited in the ipsilateral hippocampus, no CTGF protein expression was observed in non-lesioned hippocampus [16]. However, in the current investigation, using an open-skull weight-drop injury, which is considered to cause less learning and memory deficits and produce less degenerating neurons compared with kainic acid-induced brain injury model [17], the accumulation of non-neuron CTGF^+^ cells was not only in the direct vicinity of the lesion core, but also in cells with astrocyte phenotypes which located on non-lesioned areas.

Accumulation of CTGF^+^ astrocytes is a well-established response to traumatic brain injury, which contributes to wound healing and glial scar formation, and is beneficial for TBI recovery [16]. Reactive CTGF^+^ cells with astrocyte phenotypes accumulated rapidly after TBI. In 6-h p.i., CTGF^+^ cells with astrocyte phenotypes accumulated in the direct vicinity of the lesion core in the cortex, while as early as 6 h p.i., CTGF^+^ cells with parenchymal astrocyte phenotypes was detected in the non-lesioned areas. These results suggest a participation of CTGF in the pathological features within the astrocytic compartment. Glial/collagen scarring is composed of dense packed reactive astrocytes and matrix protein deposition sealed by formation of a new basal lamina. Hence, the restricted expression and accumulation of CTGF in TBI adds convincing evidence for the participation of CTGF in the early astrocytic CNS injury response as a key molecule contributing to TBI recovery.

## Conclusion

We have found an early accumulation of CTGF^+^ cells in open-skull weight-drop-induced TBI. Our study suggests that CTGF expression might define a subtype of activated astrocytes with a specific role in the tissue-repair processes following TBI.

## Abbreviations

CTGF: Connective tissue growth factor; TBI: Traumatic brain injury; CNS: Central nervous system; TGF-β: Transforming growth factor.

## Competing interests

The authors have no financial and commercial competing interests.

## Authors’ contributions

ZRZ pervised the conduction of the whole project, and designed. YQL, ZYZ contributed to the establishment of TBI model in rats, performed the experiments and drafted the manuscript; XML, BWL and XFY analysed data and did part of the animal study, sample collection and measurement; ZYZ performed the staining experiments in German; YQL and ZRZ contributed to revise the manuscript; HJS offered great advises during the whole process of this project; All authors read and approved the final manuscript.
